# Feasibility and Effects of a Gait Assistance and Gait Resistance Training Program Using a Walking-Assist Wearable Robot for Community-Dwelling Older Adults: Single-Group, Pre-, and Posttest Study

**DOI:** 10.2196/58142

**Published:** 2025-05-26

**Authors:** Eunhee Cho, Sinwoo Hwang, Seok-Jae Heo, Bokman Lim, Jewoo Lee, Younbaek Lee

**Affiliations:** 1 Mo-Im Kim Nursing Research Institute, College of Nursing, Yonsei University, Seoul, Republic of Korea; 2 College of Nursing, Graduate School, Yonsei University, 50-1 Yonsei-ro, Seodaemoon-gu, Seoul, 03722, Republic of Korea, 82 10-3721-7432; 3 Army Cadet Military School, Goesan, Republic of Korea; 4 Biostatistics Collaboration Unit, Department of Biomedical Systems Informatics, College of Medicine, Yonsei University, Seoul, Republic of Korea; 5 WIRobotics Incorporation, Youngin, Republic of Korea

**Keywords:** older adult, exercise, wearable robot, walking speed, physical functional performance, muscle strength

## Abstract

**Background:**

Two-thirds of people aged 65 years and older may require help with daily activities such as eating, bathing, and getting in and out of bed or a chair. Walking-assist wearable robots have shown significant improvements in physical function in controlled settings for patients.

**Objective:**

In this study, we aimed to assess the feasibility and the effect of a gait assistance and gait resistance training program using a walking-assist wearable robot for community-dwelling older adults.

**Methods:**

A total of 23 community-dwelling older adults aged 65 years and older (30 participants recruited, 7 dropped out) enrolled in a 12-session, 6-week gait assistance and gait resistance training program using a walking-assist wearable robot. A single-group, pre- and posttest design was employed to evaluate the feasibility based on program adherence and effectiveness. The primary and secondary outcomes for evaluating effectiveness were walking speed and functional performance, respectively.

**Results:**

Regarding the feasibility, the average number of sessions attended was 11.7 out of 12, indicating a mean adherence rate of 97.8%. Linear mixed model analysis revealed significant improvements in walking speed and functional performance at the end of the program compared with baseline. Specifically, the walking speed measured using the 10-Meter Walk Test, which includes self-selected velocity and fastest safe velocity, improved by a mean of 0.15  (SD 0.13) m/s (*P*<.001) and 0.15 (SD 0.17) m/s (*P*<.001), respectively. Functional performance also improved, with faster performance in Timed Up-and-Go (mean −0.63, SD 0.92 s; *P*=.003) and Four Square Step Test (mean −1.71, SD 1.64 s; *P*≤.001). Leg muscle strength increased across all measured domains, including plantarflexion (mean +7.29, SD 4.92; *P*=.004), hip adduction (mean +3.03, SD 2.73; *P*≤.001), hip extension (mean +2.63, SD 2.50; *P*≤.001), knee extension (mean +2.33, SD 3.12; *P*≤.001), knee flexion (mean +2.19, SD 2.17; *P*≤.001), dorsiflexion (mean +2.10, SD 3.06; *P*≤.001), hip abduction (mean +1.59, SD 1.92; *P*=.002), and hip flexion (mean +0.90, SD 1.56; *P*≤.001).

**Conclusions:**

This study stands out for applying gait assistance and resistance training across various terrains, unlike previous studies that only tested gait assistance in controlled environments. The results demonstrated significant improvements in walking speed and functional performance in older adults, suggesting the effectiveness of preventive health care services using a walking-assist wearable robot as an intervention that can contribute to improving independent functioning and frailty among community-dwelling older adults.

## Introduction

### Background

Global life expectancy has gradually increased from 66.8 years in 2000 to 73.4 years in 2019, although health-adjusted life expectancy (HALE) was only 63.7 years in 2019 (86.3% of life expectancy) [1]. In other words, HALE significantly lags behind life expectancy, with an increase in HALE (5.4 y) failing to keep up with the increase in life expectancy (6.6 y). This implies that people are experiencing prolonged periods of poor health in their older years. In particular, age-related declines in physical motor function can lead to limitations in activities of daily living (ADL) such as dressing, washing, getting out of bed, and using the restroom [2]. According to a recent report by the World Health Organization [3], approximately two-thirds of people aged 65 years and older may require help with daily activities such as eating, bathing, and getting in and out of bed or a chair. Among those aged 90 years and older, 96% may require assistance to maintain their daily functioning.

As awareness of the importance of physical activity for older adults increases, the US Department of Health and Human Services, which oversees the Office of Disease Prevention and Health Promotion [4], annually manages the physical activity rate of older adults with physical or cognitive health problems as a Healthy People 2030 indicator. In 2018, 41.3% of older adults were found to be physically active, and the target was set at 51%, which is managed as a national health policy indicator. Physical independence is primarily centered on gait. As older adults age, they tend to experience weakened lower extremity muscle strength and shorter stride lengths, resulting in slower walking speeds and difficulty maintaining balance and stability, which can lead to falls [5]. Furthermore, decreased walking speed is associated with functional loss and mortality, and is frequently used as not only a predictor of fall risk and disability but also an indicator of frailty in older adults [6]. Regular physical activity plays an important role in maintaining the health and independent functioning of older adults and has been linked to improvements in cognitive impairment and mental health. Previous studies [7-9] have shown that physical inactivity in older adults is a major contributor to the development of disuse syndrome, a vicious cycle that ultimately leads to disability in performing ADL if appropriate interventions are not staged.

Assist robots are recognized as a revolutionary technology that significantly enhances physical activity capabilities and provides rehabilitation or retraining opportunities for people with limited mobility due to injury. Wearable robots have been used to improve muscle strength in the general population; assist workers with mobility in industries such as construction, firefighting, or the military; and assist and rehabilitate people with gait weakness [10]. Unlike conventional robots, the latest commercialized robots can be worn and used to assist walking or enhance muscle strength based on the wearer’s posture information and gait data. Wearing a robot can increase walking efficiency by more than 7% [11]. Furthermore, it can be used without restrictions when sitting in a chair or walking up and down stairs.

Previous studies evaluating the effects of training with walking-assist wearable robots in older adults have reported improvements in physical function, including increased walking speed and stride length [11-14] and increased muscle strength [13,15]. These findings suggest that physical activity promotion programs using walking-assist wearable robots can be extended to older adults in the community, although they have mostly been used for rehabilitation of patients with spinal cord injury [12], multiple sclerosis [16,17], and stroke [13]. A lack of research on preventive care for older adults remains. While some studies have targeted the general older adult population, they have involved single interventions in controlled laboratory settings, limiting their applicability to community settings [11,18]. Improving the HALE of community-dwelling older adults necessitates further studies that can evaluate the effectiveness of gait assistance training in a systematic physical activity promotion program. Progressive resistance training has been shown to positively impact muscle strength and functional limitations in older adults [19]. Thus, maximizing the effect of improving older adults’ functional mobility and functional performance by applying the gait resistance function of wearable robots may not only assist gait but also strengthen muscle strength. Confirming the value of using walking-assist wearable robots to enhance walking speed and functional performance of community-dwelling older adults in the aging era may contribute to a preventive strategy that helps maintain independent physical activity while also improving HALE in the future.

In this study, we conducted a 6-week physical activity program incorporating gait assistance and gait resistance training for community-dwelling older adults using a walking-assist wearable robot, aiming to assess the feasibility and the effect on walking speed and functional performance, and to evaluate the validity of the intervention approach.

### Purpose

The purpose of this study was to identify the feasibility and effects of a gait assistance and gait resistance training program using a walking-assist wearable robot on walking speed and functional performance for community-dwelling older adults. The primary outcome of this study was walking speed, which served as the main indicator of functional mobility. Secondary outcomes included comprehensive evaluation of the participants’ functional performance, such as the Short Physical Performance Battery, Timed Up-and-Go, Four Square Step Test, Functional Reach Test, and measures of muscle strength.

## Methods

### Study Design and Setting

This study used a single-group, pretest-posttest design to determine the feasibility of program adherence and evaluate the effectiveness of a gait assistance and gait resistance training program using a walking-assist wearable robot on community-dwelling older adults. It was conducted in collaboration with a community service center and a senior living community to recruit community-dwelling older adults.

### Participants

The participants were older adults living in the community, aged 65-90 years old, who were able to walk independently for more than 30 minutes, who understood the contents of the study, and who voluntarily agreed to participate. For the purpose of this study, it was essential for participants to be able to wear a wearable robot and undergo training. Thus, the specific exclusion criteria were as follows. First, individuals with difficulty walking due to vision defects, fractures, and so on. Second, individuals with body types that prevent them from wearing the robot, such as a height of 4.59 feet (140 cm) or less or 5.91 feet (185 cm) or more, or severe obesity with a BMI of 35 or more. Third, individuals with heart and circulatory conditions that may affect walking training. Fourth, individuals at a high risk of falling during walking training, owing to severe dizziness and having experienced a fall within the preceding 2 months.

The sample size was calculated using the G*Power 3.1 program for a paired *t* test with a significance level of *P*=.05, power of 0.80, and effect size of 0.59 [20], specifically based on the primary outcome of this study, which is walking speed. The minimum sample size required was 25, and a total of 30 participants underwent convenience sampling to accommodate an approximate 20% dropout rate. During the 6 weeks of the program, 7 participants withdrew from the study for reasons such as lagging behind in pace and feeling overwhelmed by the group exercise, leaving 23 participants in the final analysis.

### Gait Assistance and Gait Resistance Training Program

**Figure 1.**
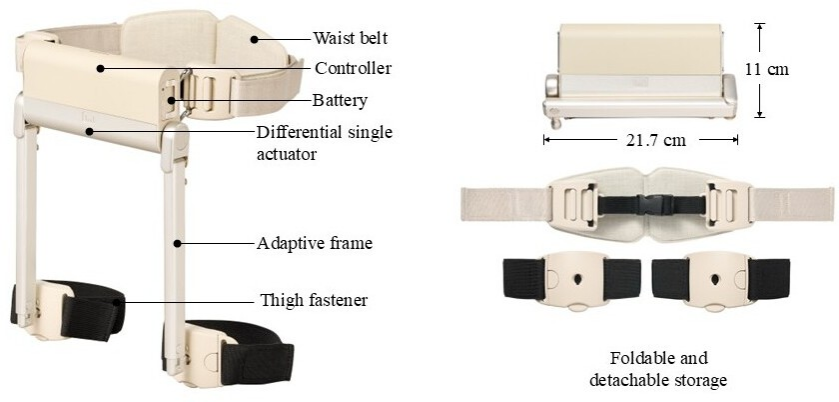
The wearable robot and its components.

The walking-assist wearable robot used in this study not only assists gait by recognizing the intention of the motion, thereby reducing the energy required for walking metabolism, but also strengthens muscles by adjusting the gait resistance intensity (Figures1  2). The wearable robot (WIRobotics Inc) is ultralightweight at 1.6 kilograms, easy to wear, and can detect postures such as walking, climbing or descending stairs, and sitting. Its body-compatible articulation structure does not limit the ability to change positions, such as sitting or lying down [21]. Unlike conventional robots, the main body and actuation parts are not located at the hip joints and back to avoid restricting body movements and to facilitate movement even in cramped spaces. In addition, the device is portable, lightweight, easy to use, and cost-effective, as it uses a single actuator, unlike conventional dual-actuator motors. Korea certification and electromagnetic compatibility tests have been completed, confirming the device’s safety and reliability.

**Figure 2.**
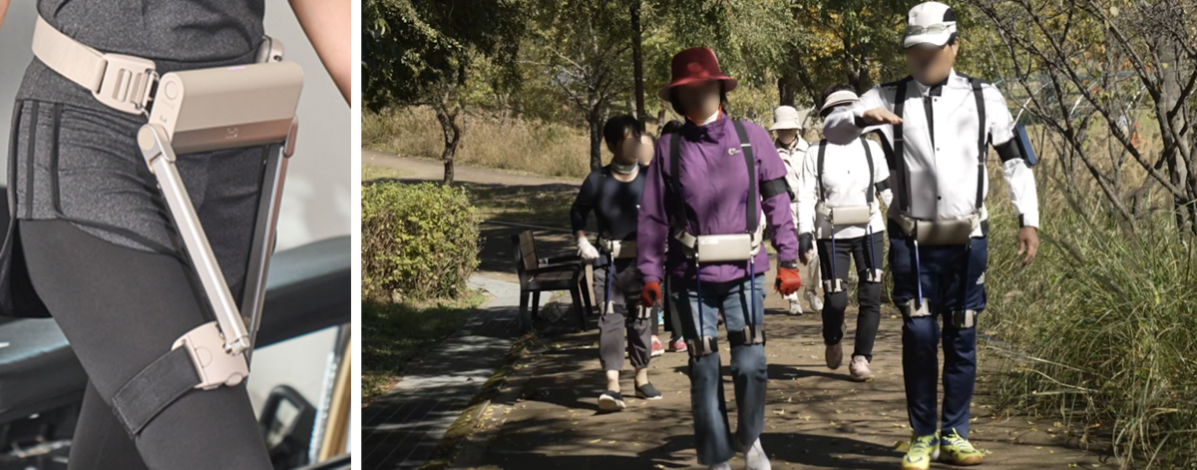
Wearing appearance and application of the wearable robot in an outdoor walking group exercise.

The gait assistance and gait resistance training program lasted 6 weeks and consisted of biweekly group exercise sessions. The gait assistance mode can be configured at 3 intensity levels, with level 1, level 2, and level 3 corresponding to peak assistance torques of approximately 4.0 Nm, 5.0 Nm, and 6.0 Nm, respectively. The intensity was set to the highest comfortable level for each participant, with level 2 being the most commonly used during the exercise sessions. During gait assistance, the device provides synchronized hip flexion and extension assistance, enhancing the wearer’s natural gait by applying positive power transfer from the device to the wearer. Conversely, the gait resistance mode functions in opposition to the assistance mode, applying resistance to hip movements that counteract the wearer’s gait. This mode facilitates negative power generation, where power is transferred from the wearer to the device, thereby increasing the difficulty of walking, like moving through water. The resistance levels are also adjustable, with peak torques of 1.5 Nm, 2.5 Nm, and 4.0 Nm for level 1, level 2, and level 3, respectively. The robot’s interface is designed for ease of use, allowing mode transitions—such as assistance, resistance, or rest (0 torque)—with a single button press. Intensity adjustments within each mode can be managed by double-pressing the button, cycling through level 1 to level 3. This user-friendly control enables participants to conveniently modify assistance or resistance levels during training sessions, following guided instructions, and facilitates engagement in group exercise activities. The first 3 weeks focused on improving walking speed and correcting walking posture using only the wearable robot’s gait assistance function, while the fourth week included 10 minutes of walking exercises with added gait resistance, increasing by 5 minutes per week to 15 minutes in the fifth week and 20 minutes in the sixth week. Participants could self-regulate the intensity of gait resistance and complete the program at a lower intensity if the exercise intensity was too much for them.

Each session of the program consisted of 10 minutes of warm-up exercises, 40 minutes of walking exercises while wearing the walking-assist wearable robot, and 10 minutes of cool-down exercises after removing the robot, totaling 1 hour. Before the training program, participants’ blood pressure, blood glucose, and physical abnormalities (skin damage, joint pain, muscle pain, etc) were checked by a nurse and physiotherapist at the institution. If no abnormalities were detected, the researchers helped the participants wear the walking-assist wearable robots and proceeded with the training program, which involved walking on flat surfaces, inclines, and stairs. The exercise was conducted in settings such as parks and walking paths within senior residential areas, covering various terrains including flat paths, slopes, and staircases. The exercise intensity was based on the speed at which the participant could walk for 40 minutes, with instructions to maintain a natural walking pace and stride.

### Feasibility (Program Adherence)

Feasibility was assessed based on program adherence, which was calculated as the number of sessions attended out of the total 12 exercise sessions for each participant.

### Effectiveness Evaluation: Primary Outcome (Walking Speed)

The 10-Meter Walk Test (10MWT) comprises 2 components—walking at normal speed (10MWT for self-selected velocity) and walking at maximum speed (10MWT for fastest safe velocity). It involves measuring the time taken to walk 10 meters and calculating the time taken to walk the middle section of 10 meters, excluding the 2.5 meters of acceleration and deceleration at the beginning and end. These times are then converted to speed (m/s). A higher distance per second after conversion indicates a faster walking speed. Intra- and interrater reliability were 0.88 and 0.99, respectively [22].

### Effectiveness Evaluation: Secondary Outcomes

#### Short Physical Performance Battery

The Short Physical Performance Battery (SPPB) is a tool developed in a multicenter study conducted by the National Institute of Aging in the United States [23]. It involves a brief physical performance test that assesses lower extremity function and includes 3 components—upright balance test, walking speed, and getting up from a chair. Each task is assigned a score of 0 to 4 [23]. The upright balance test consists of a side-by-side stance, a semitandem stance, and a tandem stance, each of which is tested in sequence and assessed by the examiner. Each stance is to be maintained for at least 10 seconds after being demonstrated to the participant. The walking speed item asks the participant to walk 4 meters at their normal walking speed, and the time taken to walk is measured. The getting up from a chair test is evaluated by measuring the time it takes to stand up and sit down from a chair 5 times with arms crossed in front of the chest. A higher SPPB total score indicates better physical performance.

#### Timed Up-and-Go

The Timed Up-and-Go (TUG) is a get-up-and-walk test to assess dynamic balance and mobility. It measures the time it takes to get up from a chair, walk 6 meters round trip, and sit down. The shorter the time (sec) taken to complete the TUG test, the better the dynamic balance and mobility. It has a reported intra- and interrater reliability of 0.99 and 0.98, respectively [24].

#### Four Square Step Test

The Four Square Step Test (FSST) is a quadrant step test that uses 4 cylindrical rods 90 cm long and 1 cm in diameter to create a cross-shaped square on the floor. It measures the time taken for the participant to stand in square 1 and then move as quickly as possible clockwise to 2 (forward), 3, 4 (backward), and 1, and then counterclockwise to 4, 3, 2, and 1 [25]. The shorter the time taken (sec) to complete the FSST test, the higher the level of dynamic balance and mobility. It has a reported intra- and interrater reliability of 0.83 and 0.99, respectively [26].

#### Functional Reach Test

The Functional Reach Test (FRT) assesses balance ability. It measures the distance participants can reach with their arms maximally extended in front of them, with a reported interrater reliability of 0.98 [27]. A longer distance (cm) measured with the arm outstretched in the FRT test indicates better dynamic balance ability.

#### Muscle Strength

Muscle strength was measured at the hip, knee, and ankle using a digital dynamometer, micro FET2 (Hoggan Scientific; range 0‐300 lb). Strength was measured in a seated and supine position using a bed, and 8 items related to strength were measured in hip flexion, hip extension, hip adduction, hip abduction, knee flexion, knee extension, dorsiflexion, and plantarflexion according to a standardized protocol. Higher values indicated greater strength of the muscle.

### Data Collection

The program intervention spanned 6 weeks, from September 20 to October 27, 2023, at the community service center and from September 18 to October 25, 2023, at the senior living community. A preassessment of primary and secondary outcomes was conducted before the program, and a postassessment was conducted after. The pre- and postassessments were collected by the same researcher.

### Ethical Considerations

This study was conducted after receiving approval from the Public Institutional Review Board of the National Center for Bioethics Policy (P01-202309-01-032). Interested participants were recruited from the community service center and the senior living community in Gyeonggi-do with previous approval of the managers and the cooperation of visiting nurses and physiotherapists in these institutions. Those who were willing to participate in the study were asked to contact the research director, who explained the purpose, method, and confidentiality in detail, first over the phone and then in person. Written consent (Multimedia Appendix 1) was obtained from all participants, an orientation session was conducted on how to use the wearable robot, and the participation schedule was coordinated. After participating in the program, light refreshments (such as water and bread valued at less than US $7) were provided to support hydration and energy replenishment. Participants who completed the training program were given a certificate and a small gift valued at approximately US $14 as appreciation for their participation in the study. Research data were stored in a place accessible only to the researcher, and electronic files were encrypted to protect the rights of research participants. Furthermore, all participant data were fully anonymized to prevent the identification of individuals. Consent to publish their image has been obtained from all individuals pictured in Figure 2.

### Statistical Analysis

The general characteristics of the participants are presented as mean (SD) for continuous variables or number (percentage) for categorical variables. Comparisons of mean differences in the primary and the secondary outcome before and after the program were analyzed using paired *t* tests. The normality of the pre-post differences was assessed using histograms and Q-Q plots. While the SPPB scores exhibited some deviations from a normal distribution, their approximately symmetrical pattern supported the use of a parametric test (paired *t* test) for the analysis. We also used linear mixed models to adjust for baseline value, gender, age, and BMI while accounting for repeated measures. Furthermore, 2-sided *P* values less than .05 were considered statistically significant. All statistical analyses were performed using SAS version 9.4 (SAS Institute Inc) and R software (version 4.2.0; R Foundation for Statistical Computing).

## Results

### General Characteristics of the Participants and Feasibility

The general characteristics of the 23 participants are presented in Table 1. Their mean age was 75.87 (SD 4.70) years. They included 18 (78%) women and 5 (22%) men. Their mean height was 156.00 (SD 10.69) cm, mean weight was 59.20 (SD 10.69) kg, and mean BMI was 24.20 (SD 3.32). Regarding the feasibility, the mean adherence rate was 97.8%, with participants attending an average of 11.7 out of 12 exercise sessions (range 10‐12 sessions). The reasons for missed sessions were primarily scheduling conflicts with personal appointments (n=4), followed by confusion regarding the session time or location (n=2).

**Table 1.** General characteristics of study participants (N=23).

Variables and categories		Statistical value
Sex, n (%)		
	Male	5 (22)
	Female	18 (78)
Age (y), mean (SD)		75.87 (4.70)
Height (cm), mean (SD)		156.00 (10.69)
Weight (kg), mean (SD)		59.20 (10.69)
BMI (kg/m^2^), mean (SD)		24.20 (3.32)

### Effects of the Training on Community-Dwelling Older Adults

The results of the linear mixed model analysis adjusted for baseline values, gender, age, and BMI are presented in Table 2. The mean walking speed and functional performance of the participants were found to improve at the end of the program compared to the baseline values. Paired *t* tests showed statistically significant improvements in all posttests except SPPB and FRT.

**Table 2.** Pre- and posttest differences of the training program on community-dwelling older adults (N=23).

Variables		Pretest, mean (SD)	Posttest, mean (SD)	Difference, mean (SD)	Paired *t* test		Linear mixed model	
					*t* test (*df*)	*P* value	Standardized β coefficient (95% CI)	*P* value
Primary outcomes								
	10MWT-SSV^a^ (m/s)	1.48 (0.16)	1.33 (0.16)	0.15 (0.13)	−5.69 (22)	<.001	0.15 (0.10 to 0.20)	<.001
	10MWT-FV^b^ (m/s)	1.76 (0.21)	1.61 (0.20)	0.15 (0.17)	−4.29 (22)	<.001	0.15 (0.09 to 0.22)	<.001
Secondary outcomes								
	SPPB^c^ (score)	11.74 (0.61)	11.96 (0.20)	0.22 (0.67)	1.55 (22)	.14	0.22 (0.00 to 0.44)	.05
	TUG^d^ (sec)	8.30 (1.32)	7.67 (1.29)	−0.63 (0.92)	−3.30 (22)	.003	−0.63 (−1.02 to −0.25)	.002
	FSST^e^ (sec)	9.33 (2.21)	7.62 (1.18)	−1.71 (1.64)	−4.99 (22)	<.001	−1.71 (−2.29 to −1.13)	<.001
	FRT^f^ (cm)	30.37 (5.68)	32.09 (5.10)	1.72 (5.58)	1.48 (22)	.15	1.72 (−0.50 to 3.94)	.13
Muscle strength								
	Hip flexion	12.59 (3.71)	13.49 (3.23)	0.90 (1.56)	2.79 (22)	.01	0.90 (0.31 to 1.50)	.004
	Hip extension	12.40 (2.38)	15.03 (3.15)	2.63 (2.50)	5.05 (22)	<.001	2.63 (1.70 to 3.56)	<.001
	Hip adduction	7.57 (1.93)	10.60 (3.70)	3.03 (2.73)	5.33 (22)	<.001	3.03 (2.02 to 4.04)	<.001
	Hip abduction	8.19 (1.97)	9.78 (2.96)	1.59 (1.92)	3.98 (22)	<.001	1.59 (0.84 to 2.35)	<.001
	Knee flexion	13.00 (3.36)	15.19 (2.25)	2.19 (2.17)	4.84 (22)	<.001	2.19 (1.40 to 2.98)	<.001
	Knee extension	15.17 (4.01)	17.50 (3.49)	2.33 (3.12)	3.58 (22)	.002	2.33 (1.07 to 3.58)	<.001
	Dorsiflexion	17.01 (3.20)	19.11 (3.83)	2.10 (3.06)	3.30 (22)	.003	2.10 (0.85 to 3.35)	.002
	Plantarflexion	15.63 (2.05)	22.93 (4.18)	7.29 (4.92)	7.11 (22)	<.001	7.29 (5.41 to 9.17)	<.001

a 10MWT-SSV: 10-Meter Walk Test for self-selected velocity.

b 10MWT-FV: 10-Meter Walk Test for fastest safe velocity.

c SPPB: Short Physical Performance Battery.

d TUG: Timed Up-and-Go.

e FSST: Four Square Step Test.

f FRT: Functional Reach Test.

Specifically, the participants’ 10MWT self-selected velocity improved by an average of 0.15 (SD 0.13) m/s, and their 10MWT fastest safe velocity improved by an average of 0.15 (SD 0.17) m/s. SPPB improved by an average of 0.22 (SD 0.67) points. TUG and FSST times decreased by –0.63 (SD 0.92) seconds and –1.71 (SD 1.64) seconds, respectively. FRT increased by 1.72 (SD 5.58) cm. All muscle strength measurements showed an increase, with the following values: plantarflexion (mean 7.29, SD 4.92), hip adduction (mean 3.03, SD 2.73), hip extension (mean 2.63, SD 2.50), knee extension (mean 2.33, SD 3.12), knee flexion (mean 2.19, SD 2.17), dorsiflexion (mean 2.10, SD 3.06), hip abduction (mean 1.59, SD 1.92), and hip flexion (mean 0.90, SD 1.56).

## Discussion

### Principal Findings

This study was conducted to assess the feasibility and the effect of a training program that uses a walking-assist wearable robot to enhance walking speed and functional performance in community**-**dwelling older adults. The program incorporated gait resistance training as well as gait assistance on flat surfaces, slopes, and stairs in community settings, which distinguishes this study from previous studies that examined the effects of gait assistance on patients in a controlled setting. The results demonstrated significant effects in improving walking speed and functional performance in older adults. These findings confirm the potential for preventive health care services using a walking-assist wearable robot to contribute to the improvement of the health status and physical frailty of community-dwelling older adults.

The participants in the program showed a significant increase in walking speed of 0.15 m/s. This is consistent with previous studies that also showed an improvement in walking speed after wearable robot–assisted gait training [12-14]. Previous research [28] has established that a change in walking speed ranging from 0.10 to 0.14 m/s represents a minimal important change with a medium effect size. Therefore, the improvement observed in this study exceeds this threshold, indicating both statistical and clinical significance. Although this was achieved over a relatively short intervention period, it suggests that extended programs could lead to even greater improvements, further enhancing participants’ mobility and quality of life. Previous studies have demonstrated that a walking-assist wearable robot can significantly reduce metabolic energy consumption during walking. For instance, using the device for walking assistance reduced energy expenditure by 15.7% on inclines and 10.6% on flat surfaces in young adults, which is equivalent to lowering the payload by approximately 9 kg [21,29]. In this study, the robot operates by applying torque directly to the hip joint, influencing the entire lower limb both directly and indirectly. In older adults, this hip-assist function, which aids in hip flexion and extension, can promote a more energy-efficient gait, enabling longer strides and sustained walking speed by reducing metabolic demand compared with unaided walking. In addition, as shown by Lenzi et al [30], hip exoskeleton assistance can decrease the activation of both proximal hip and distal ankle muscles. Given that aging is associated with decreased lower limb strength and reduced ankle propulsion, older adults tend to compensate by generating more power at the hip, resulting in about 17% higher metabolic energy consumption during walking compared with younger individuals [31,32]. Therefore, hip assistance is anticipated to enhance stride length and walking speed, helping maintain these improvements throughout extended periods. Furthermore, the findings are similar to the results of a study in which older adults were divided into 3 groups—independent walking, partially assisted by a robot, and fully assisted by a robot. The group that was fully assisted by a robot exhibited a significant increase in walking speed [11]. Decreased walking speed is a known risk factor for disability, frailty, falls, cognitive impairment, and mortality among community-dwelling older adults [6,33]. It is also a significant predictor of disability in ADL [34,35]. In view of these considerations, older adults’ increased walking speed may contribute to maintaining their daily physical activity and independent functioning by reducing future rates of disability or impairment. Therefore, training programs using wearable robots can be considered a preventive health care service for community-based aging in place, aligning with a global policy trend in the era of aging.

The results also revealed statistically significant improvements in the TUG and FSST, with the time required for each test reducing by an average of 0.63 (SD 0.92) seconds (7.59%) and 1.71 (SD 1.64) seconds (18.33%), respectively. These results are similar to previous studies that have reported improvements in TUG [12-14,18,36] and FSST [18] after training with a wearable robot. In this study, the TUG showed a reduction of 0.63 seconds, exceeding the minimal important change threshold of 0.4 seconds. This suggests a clinically significant improvement, indicating enhanced mobility and a potential reduction in the participants’ fall risk. In general, the TUG and FSST are reported to be associated with a higher risk of falling if they take longer than 15 seconds to complete [37]; community-dwelling older adults are expected to be able to perform these tests in less than 12 seconds [36,38]. Similar to walking speed, TUG and FSST in older adults have been reported to be key predictors of health deterioration, new ADL difficulties, and falls [35,39]. The results of this study highlight the importance of the use of wearable robots as a physical activity promotion interface to support independent community living in older adults.

The postintervention assessment also revealed significant improvements in leg muscle strength. These results are similar to those of previous studies on patients with impairments [15,17]. The participants’ muscle strength potentially improved as the wearable robot supported their gait efforts to move their joints. In addition, the introduction of a gait resistance mode helped address the typical drawback of walking exercises, where it can be challenging to increase muscle strength load. By allowing participants to exert more force, particularly from week 4, and gradually increasing the resistance time by 5 minutes per week, the resistance mode contributed to strength gains. Furthermore, the combination of walking training on not only flat surfaces but also inclines and stairs may have contributed to muscle strength improvements. A previous study [19] also identified progressive resistance training programs as an effective strategy for increasing muscle strength and bone formation in older adults aged more than 75 years. Although this study used progressive training with gradually increasing gait resistance intensity, differences in the effectiveness of resistance training may exist among participants, as they were allowed to self-regulate the intensity to complete the exercise. Therefore, programs should ideally include a progressive training method customized to the individual’s condition to improve muscle strength without causing muscle fatigue or injury.

Although the SPPB and FRT showed improvements in this study, they were not statistically significant. Regarding the SPPB, our study observed an improvement of 0.22 points. While this change did not reach the established clinical threshold of 0.54 points [28] typically required for a meaningful improvement, it is important to consider the high baseline performance of our participants, who had scores close to the maximum possible. The SPPB is a 12-point test, on which the participants scored an average of 11.74 (SD 0.61) in the pretest, indicating that most of them were close to perfect, which may explain the lack of statistical significance due to the small variation and ceiling effect [40]. Ceiling effects in highly functional groups may limit the SPPB’s sensitivity to detect subtle changes. Future studies should consider using more sensitive assessment tools to better capture subtle improvements in similar populations. The FRT is a test that reflects dynamic balance ability [41]; while a previous study [14] has reported significant effects, this study’s results differed.

However, slight improvements in SPPB and FRT suggest that the program may have improved functional performance in older adults.

In addition, walking speed and functional performance, which were statistically significantly improved by paired *t* tests, remained statistically significant after adjusting for the baseline values, gender, age, and BMI, confirming the program’s effectiveness. From the perspective of intrinsic capacity, a core element of the World Health Organization’s framework on healthy aging [42], it is essential to maintain and enhance physical capacities even in individuals with high baseline function. The program’s effectiveness in preserving mobility, strength, and overall functional performance underscores its potential to support healthy aging by fostering the sustained development of intrinsic capacity, which is crucial for long-term functional ability and independence. Furthermore, future research should target more frail populations, as they may experience even greater benefits from such interventions. This would allow for a clearer assessment of the program’s potential to improve intrinsic capacity and functional ability in individuals with lower baseline performance, addressing a broader range of needs within the aging population.

### Limitations

This study has several limitations. First, the absence of a control group and the lack of a postrepeat measurement limit the elucidation of the program’s direct and residual effects, thus making it difficult to establish causality. Therefore, the long-term effects should be confirmed with a control group in future studies to address these limitations. Second, the evaluators who conducted both pre- and postassessments were not blinded. While this arrangement may introduce potential bias, we attempted to minimize subjective influence by using objective evaluation tools, such as walking speed and muscle strength measurements. Future studies should consider using independent, blinded evaluators to further reduce the risk of bias and ensure greater objectivity in outcome assessments. In addition, in cases where physical function is relatively high, as observed in the participants of this study, the SPPB may have limitations in detecting subtle changes. This can lead to difficulties in achieving statistical significance due to a ceiling effect, where participants score near the maximum limit, reducing the sensitivity of the measure. Therefore, future studies should consider using more sensitive and appropriate assessment tools that can accurately capture subtle variations, thereby improving the reliability of the findings. Finally, during the 6 weeks of the program, 7 participants dropped out because of the burden of group exercise, lowering the statistical power of the study, as only 23 participants in total were analyzed. In group training, a wide gap in pace with other people tends to be psychologically discouraging, making it difficult to continue exercising. It may be advisable to conduct group training in small groups of no more than 3‐4 people, considering individual physical abilities. Nonetheless, the significant walking speed and functional performance improvements revealed in this study support the effectiveness of the program using a walking-assist wearable robot for community-dwelling older adults.

### Conclusions

In conclusion, implementing a preventive intervention of an appropriate physical activity program using a wearable robot proved effective in improving walking speed and functional performance in community-dwelling older adults. This study provides evidence of its feasibility and effectiveness as an intervention to promote independent and successful aging in older adults. In addition, our findings confirm the clinical potential of the gait assistance and gait resistance training program using a wearable robot as a preventive health care service for community-dwelling older adults in an aging society.

## Supplementary material

10.2196/58142 Multimedia Appendix 1 Informed consent form.

## Abbreviations

10MWT: 10-Meter Walk Test
ADL: activities of daily living
FRT: Functional Reach Test
FSST: Four Square Step Test
HALE: health-adjusted life expectancy
SPPB: Short Physical Performance Battery
TUG: Timed Up-and-Go
