# Supplementary material for: Feasibility and Effects of a Gait Assistance and Gait Resistance Training Program Using a Walking-Assist Wearable Robot for Community-Dwelling Older Adults: Single-Group, Pre-, and Posttest Study
Source: JMIR Mhealth Uhealth. 2025 May 26;13:e58142. doi: 10.2196/58142 (PMC12129416; doi:10.2196/58142)

Ministry of Health and Welfare Designated Public Institutional Review Board [Recommendation Form No. 2-1]

**Consent ver.2.0**

**Research Title: Walking Assistance and Resistance Program Using Ultralight Wearable Robots for Older Adults**

1. I have read the description of this study and have discussed it with the researcher in charge.
2. I have been informed of the risks and benefits and have received satisfactory answers to my questions.
3. I voluntarily agree to participate in this study.
4. I agree that information about me obtained in this study will be collected and processed by the researcher within the scope permitted by current laws and institutional review board regulations.
5. I agree to allow the principal investigator or an authorized delegate to access my personal information, as well as the research institution, funding agency, and the public institutional review board designated by the Ministry of Health and Welfare for the purposes of monitoring, auditing, or managing the study, under strict confidentiality.
6. I understand that I may withdraw from this study at any time without penalty or disadvantage to myself.
7. My signature means that I have received a copy of this consent form and I will keep a copy until the end of my participation in the study.

| Participant | Name: | Signature: | Date of Signature: |
| --- | --- | --- | --- |
| Legal Representative (if necessary) | Name:  Relationship to the Participant: | Signature: | Date of Signature: |
| Observer  (if necessary) | Name: | Signature: | Date of Signature: |
| Person Obtaining Consent | Name: | Signature: | Date of Signature: |


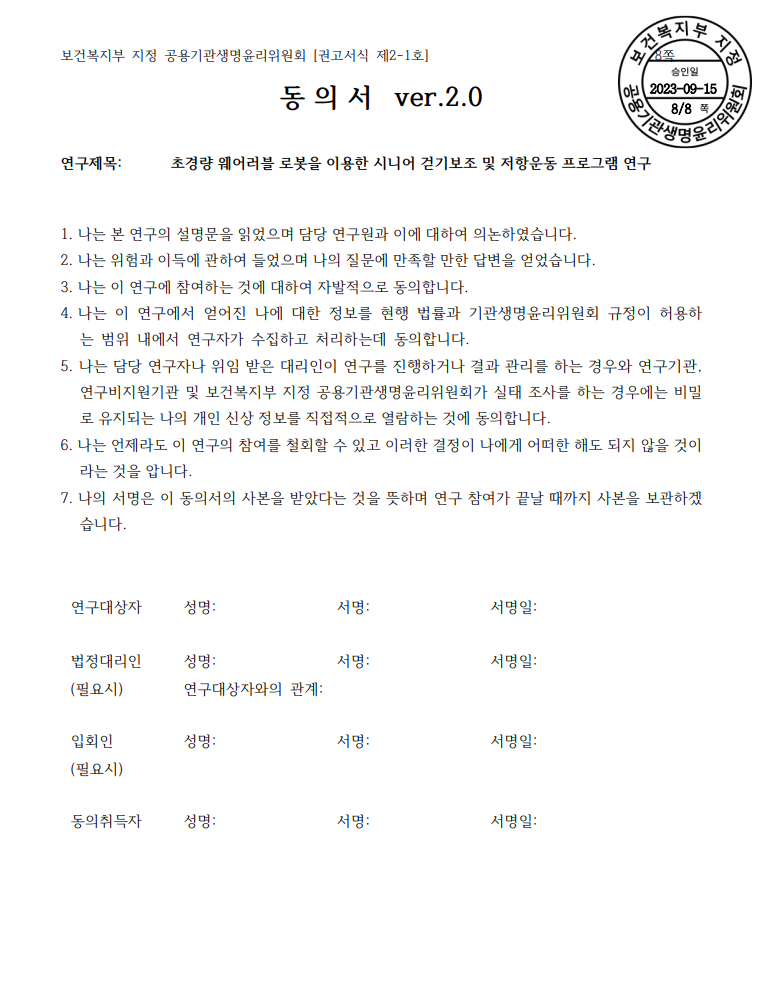

Supplement: Multimedia Appendix 1 [file mhealth-v13-e58142-s001.docx]
